# Is Pharmacy a Friendly Profession for Everyone in the U.S.? A Probe into Gender and Other Income Disparities

**DOI:** 10.3390/pharmacy13020049

**Published:** 2025-03-26

**Authors:** Ioana Popovici, Manuel J. Carvajal

**Affiliations:** Department of Sociobehavioral and Administrative Pharmacy, Barry and Judy Silverman College of Pharmacy, Nova Southeastern University, 3200 South University Drive, Fort Lauderdale, FL 33328-2018, USA; cmanuel@nova.edu

**Keywords:** earnings, gender gap, human capital, job-related preferences, labor input, pharmacist workforce

## Abstract

The literature shows that women persistently earn less than men for similar roles and qualifications; yet, pharmacy has been portrayed as an egalitarian profession, with a small gender earnings gap relative to other occupations. There is a lack of recent studies, and some evidence suggests a significant gender gap when earnings are estimated separately for male and female pharmacists. This study compared the nature and magnitude of gender income disparities using two alternative methodological procedures and evaluated the evidence for policy implications. The study was based on 2019–2022 American Community Survey (ACS) data collected by the U.S. Census Bureau. The sample consisted of 12,450 pharmacists (61.5% women) ages 25–64 years practicing in the U.S. Ordinary least-squares models calculated pharmacist annual incomes as functions of work input, human-capital, and job-related covariates. Results estimated a gender earnings gap of up to 18.6%. Differences across sociodemographic groups suggested that pharmacy is only friendly to selected segments of the profession. The empirical evidence reported here is expected to be used by healthcare managers and policymakers to inform ongoing discussion regarding the need for policy changes and cultural shifts to promote gender equity.

## 1. Introduction

Numerous intra-occupational income studies have identified disparities between genders, indicating that women earn less than men for similar roles and qualifications [1,2,3,4]. These disparities usually are attributed to factors such as societal expectations and stereotypes about gender roles, dissimilar advancement opportunities, different negotiation practices, heterogeneous job-related preferences, discrimination in the workplace, and women working on average fewer hours than men. Presumably society’s perception of women as the main caregivers and responsible for domestic duties lead to more interruptions in their careers and fewer hours worked both in the short and long run [5]. As a result, women are less likely to be promoted and earn lower wages and salaries than men [6,7]. Moreover, men generally gravitate toward jobs that highlight monetary aspects such as salary, overtime, and promotion potential, whereas women are more inclined to pursue positions that offer flexible schedules, positive coworker interactions, and other non-financial benefits [8]. The literature has documented the persistent nature of this gap, prompting ongoing discussion regarding the need for policy changes and cultural shifts to promote gender equity [9,10].

While gender income disparities are found virtually everywhere, pharmacy has been portrayed in seminal research by Goldin and Katz [11] as an egalitarian profession characterized by a modest gender pay gap and less earnings variation compared to other occupations. The authors argued that substitutability among pharmacists due to uniform training, standardization of products, technological change, and growth of pharmacy employment in retail chains and hospitals have contributed to narrowing the gender income gap in pharmacy over time. Their study concluded that pharmacists’ earnings were largely proportional to the number of hours worked.

Conversely, in a study of pharmacists in South Florida, Carvajal et al. [12] reached different conclusions. They found that, beyond the number of hours worked, pharmacists’ earnings also were determined by factors such as opinion variables and professional roles. They argued that male and female earnings were influenced by different factors, and when earnings were determined by the same variables, the effect often was dissimilar. After accounting for number of hours worked, human capital, and job preferences, their study found that male pharmacists earned higher wages and salaries than their female counterparts, highlighting a significant gender pay gap.

In light of these seemingly mixed findings, the current probe was aimed at revisiting the topic using recent data (2019–2022) from the largest household survey in the United States and building on the work of Goldin and Katz [11] by extending their analyses to uncover pay gaps along several other dimensions such as marital status, age, race and ethnicity, sector, and employment status. Specifically, this study sought to (i) estimate the nature and magnitude of gender and other pharmacists’ income disparities, if they exist, using two alternative methodological procedures; (ii) compare the empirical findings of the alternative procedures; and (iii) evaluate the evidence for policy implications.

## 2. Methods

The methodology of this article consisted of formulating and estimating, using ordinary least-squares, two models expressing pharmacists’ income as functions of work input and selected human-capital and job-related covariates. The first model (Model 1) consisted of one equation, whereas the second model (Model 2) had two equations. Model 1 was the equation designed by Goldin and Katz [11] and examined gender as a dichotomous variable, while Model 2 was designed to estimate income for men and women separately. The differences between these models were the analysis datasets and an additional gender dichotomous variable in Model 1. Implicit in Model 1 was the assumption that the responses to covariates were equal for pharmacists of both genders [4,13]; gender-related changes in income may be measured only by assigning alternative values to the dichotomous covariate. If the effects of one or more covariates differ significantly between men and women, the one-equation model may obscure these differences. Furthermore, if gender-specific systemic biases are not captured in the equation, the overall effect of gender may be underestimated.

The two-equation model allowed for the estimation of male pharmacists’ income separately from female pharmacists’ income. Since the covariates were identical, the coefficient estimates’ direction, magnitude, and statistical significance can be compared to assess their influence on earnings while accounting for all other covariates, thereby minimizing gender disparities that might arise from interaction effects. This approach has been used successfully by Carvajal et al. [12] and Carvajal and Popovici [14] with pharmacists, and by Carvajal et al. [15] with health economics, outcomes research, and market access professionals.

**Model** **1.** *Single Equation with a Dichotomous Covariate for Gender*.

The equation for this model was specified as follows:

ln E*_i_* = α + G*_i_*β + W*_ij_*γ*_j_* + X*_ij_*λ*_j_* + Z*_ij_*θ*_j_* + u*_i_*

**Model** **2.** *One Equation for Each Gender*.

The equation for this model was specified as follows:

ln E*_i_* = α + W*_ij_*γ*_j_* + X*_ij_*λ*_j_* + Z*_ij_*θ*_j_* + u*_i_*

where

ln E*_i_* was a vector of the natural logarithm value of income earned by the *i*th pharmacist;

G*_i_* was a dummy-variable vector set to 1 if the *i*th pharmacist was a woman, 0 otherwise;

W*_ij_* was a matrix of work-input values (*j* = 2), including average number of hours worked per week and number of weeks worked during the previous year, reported by the *i*th pharmacist;

X*_ij_* was a matrix of human-capital characteristics (*j* = 8), including age and age squared, race (Black/African American, other than White or Black/African American), Hispanic ethnicity, marital status (single/never married, separated/divorced/widowed), and having at least one child, reported by the *i*th pharmacist;

Z*_ij_* was a matrix of job-related characteristics (*j* = 3), including practice setting (retail, hospital or other health services industry) and self-employment status, reported by the *i*th pharmacist;

u*_i_* was a vector of normally and independently distributed stochastic disturbance terms, with mean zero and variance σ^2^, for the *i*th pharmacist;

α was the least-squares constant term;

β was the least-squares coefficient for gender;

γ*_j_*, λ*_j_*, and θ*_j_* were vectors of *j* parameters;

and where

*i* = 1, …, n; and

n was the number of pharmacists in the sample or gender subsample.

### 2.1. Variables in Both Models

The dependent variable was defined as pharmacists’ annual, pre-tax, wage-and-salary and self-employment income adjusted for inflation, using the Consumer Price Index (CPI), to 2022 dollars. It was logged to minimize the influence of outliers and facilitate the interpretation of relative differences instead of absolute values. As a result, the estimated coefficients in the semi-log income models represented exponential values. In these models, an increase of 0.693147 units in the dependent variable would double earnings, while a decrease of 0.693147 units would halve earnings. The antilog (*e^x^*) of an estimated least-squares coefficient denotes the percentage change in income due to a one-unit change in the value of its covariate.

Selection of the independent variables in the models followed and built on the research by Goldin and Katz [11]. Work input has been the most important indicator influencing pharmacists’ wage-and salary earnings [9,11,14]. Two covariates were designed to measure work input: average number of hours worked per week and number of weeks worked during the previous year. Both covariates were expected to exert a positive effect on income.

The human-capital matrix consisted of eight covariates out of five indicators hypothesized to influence pharmacists’ income. The first indicator was age, often used as a proxy for experience. It appeared in the model with a linear and a quadratic component (i.e., two covariates) to account for diminishing returns. More years of experience were expected to yield greater productivity and higher levels of income up to a point beyond which obsolescence would occur; thus, the linear component was hypothesized to be positive and the quadratic component was hypothesized to be negative.

Race was the second human-capital indicator. Two dichotomous covariates were identified, one for Black/African American pharmacists and one for pharmacists other than White or Black/African American; White pharmacists were used as the reference group. In addition, Hispanic origin was added to the equation as a dichotomous variable (non-Hispanic origin was used as the reference group) to identify ethnicity separately from race. Several studies have found that both race and ethnicity are associated with labor market outcomes [16,17,18]. Within pharmacy, Carvajal et al. [19] have reported that racial and ethnic minority pharmacists exhibit different earnings responses to human-capital variables when compared with their non-minority counterparts. Here, the estimated least-squares coefficients for the three racial/ethnic minority covariates were expected to be negative, indicating lower earnings for minority pharmacists.

Current marital status was part of the human-capital matrix affecting earnings. Two dichotomous covariates were identified, one for single/never-married practitioners and one for separated/divorced/widowed practitioners; married practitioners were used as the reference group. Also included was a dichotomous covariate for the presence of children in the household (a value of 1 if the pharmacist had at least one child, a value of 0 otherwise). This indicator has been found to exert a negative effect on women’s earnings by virtue of limiting their participation in the workforce [20,21,22]. Thus, the least-squares coefficients for this covariate were expected to be negative for female pharmacists.

The job-related matrix consisted of three covariates out of two indicators, practice setting and self-employment status. Two dichotomous covariates were identified under practice setting, namely, working in retail pharmacy and working in a hospital or another health services industry; working in manufacturing or other settings was used as the reference group. The choice of working in alternative practice settings may be influenced not only by income, but also by the amount of job satisfaction derived from one’s professional functions. Many pharmacists express frustration that a significant portion of their time is spent dispensing medications rather than providing counseling and clinical services [23]. Dispensing is less palatable and more stressful compared to other roles [24]. Since retail pharmacy requires proportionately more time spent dispensing medications from practitioners than hospital pharmacy, pharmacists might trade off income for job satisfaction to various degrees. There is ample evidence in the literature that female pharmacists derive greater levels of both career and job satisfaction than their male counterparts [15,24,25]. This would be consistent with female pharmacists trading off income for satisfaction more often than male pharmacists, thus earning, on average, relatively lower levels of income [26]. The coefficients for the retail pharmacy covariate were expected to be higher than those for the hospital pharmacy covariate.

The last indicator considered in both models was a dichotomous covariate for self-employment. Self-employment is not for everyone; it requires a proactive, confident disposition from pharmacists willing to compete and take risks [27]. Greater risks usually are accompanied by greater rewards, so the least-squares coefficients were expected to be positive.

### 2.2. Data Source

The data for this study were drawn from the 2019–2022 American Community Survey (ACS) [28], the same data source used by Goldin and Katz [11] for 2009–2011. Each year, the U.S. Census Bureau reaches out to more than 3.5 million households nationwide to take part in the ACS. Data on social, economic, labor force, housing, and demographic characteristics are collected every month from a rotating random sample of about 250,000 addresses in the 50 states, Washington, D.C., and Puerto Rico.

The harmonized U.S. Census Bureau’s 2010 ACS occupation classification scheme was used to select pharmacists from the global survey. Only pharmacists who worked at least one week during the previous year were included. The sample for this study consisted of 12,450 pharmacists (4791 men and 7659 women) between the ages of 25 and 64 years practicing in the U.S. Their incomes were adjusted for inflation to 2022 dollars using the Consumer Price Index (CPI). The research effort was supported solely by internal university funds and Institutional Review Board’s assessment was not needed because the data were deidentified and publicly accessible. The analysis was conducted using Stata statistical package 15.0 [29].

## 3. Results

The means and standard deviations of wage-and-salary and self-employment income earned by pharmacists in the sample, as well as variables hypothesized to influence them, are presented in Table 1. The first column pertains to all pharmacists (Model 1), while the other two columns show the estimates separately for male and female pharmacists (Model 2). Women, who comprised over three-fifths of the sample, earned, on average, $19,477 annually less than men. They also worked, on average, fewer hours per week and fewer weeks per year, and were younger than male practitioners.

Two-thirds of pharmacists were White and, in the sample, proportionately there were more White men than White women. Only one out of every 20 persons reported being Black/African American (no significant gender differences) and the rest reported being from other races (higher percentage in women than in men). Fewer than one out of 20 practitioners reported being Hispanic, with no significant gender differences.

Most pharmacists (70.5%) were married—the fractions for men and women were virtually identical. Only one-fifth reported being single/never married (no significant gender differences) and the rest reported being separated/divorced/widowed; proportionately more women than men fell in the latter category. Over one-half of practitioners reported having at least one child, women reporting so more frequently than men.

In terms of job-related characteristics, more than one-half of pharmacists worked in retail pharmacy; the numbers for this category revealed a preference by male practitioners. Conversely, women seemed to prefer working in hospital pharmacy, which overall accounted for three out of every ten practitioners. The rest of the sample (14.0%) worked in manufacturing or other settings. Only a small fraction reported being self-employed, with proportionately more men than women in this category.

### Estimated Equations

The results of both models are presented in Table 2. The coefficient estimates as well as all three *F* ratios were statistically significant. In Model 2, the adjusted R^2^ value was higher for female than for male pharmacists.

According to the estimates of Model 1, female pharmacists earned 6.0% lower levels of income after work input, age, and all other covariates in the equation were considered. An additional hour of work per week yielded 2.9% higher annual income and an additional week of work per year yielded an increase of 4.0% in annual income. The effect of age, a proxy for experience, was not constant because of the quadratic term; as expected, income increased at a decreasing rate with experience. At the covariate mean, an additional year of experience brought about an average of 1.2% higher income to pharmacists.

The estimates of Model 1 also revealed that Black/African American practitioners earned 16.5% lower income levels than White practitioners, although the coefficient for practitioners whose reported cultural identity was other than White or Black lacked statistical significance. Hispanic pharmacists, however, exhibited 15.0% less than the income level reported by non-Hispanic pharmacists. Lower incomes also were experienced by single/never married (12.1% less) and separated/divorced/widowed (9.3% less) pharmacists relative to their married counterparts. The coefficient for the presence of children in the household was not statistically significant.

Working in retail pharmacy paid 4.5% less than working in manufacturing or other settings, according to the estimates of Model 1, and working in hospital pharmacy paid 4.4% more than the reference group. The last coefficient of the equation showed that, contrary to expectations, self-employed practitioners earned only 66.9% (33.1% less) of (than) the income estimated for practitioners who were not self-employed.

In Model 2, there were two separate equations with identical covariates, one for male pharmacists and the other for female pharmacists. The empirical evidence showed that for every additional hour of work per week, male practitioners earned 2.5% more income while female practitioners earned 3.2% more income. With every additional week of work per year, male and female practitioners earned 4.2% and 3.9% more income, respectively. One more year of experience, approximated by age, brought to pharmacists (at the means of the covariate) an increase in income of 1.2% for men and women alike.

Male pharmacists who were Black/African American earned 19.1% less income than their White male counterparts and Black/African American female pharmacists earned 15.2% less income than White female pharmacists. Male practitioners other than White or Black also earned less income (4.8% less) than White male practitioners, but the coefficient for female pharmacists other than White or Black lacked significance. Lower income levels, however, were experienced by Hispanic pharmacists—17.4% less for men and 13.8% less for women—relative to the rest of the sample. The income estimates also were lower for single/never-married men (9.2%) and women (14.0%), as well as for separated/divorced/widowed men (9.8%) and women (9.5%), relative to married practitioners. Neither coefficient for the presence of children in the household of male or female practitioners possessed statistical significance.

Men working in retail pharmacy earned 8.2% less income than their colleagues working in manufacturing or other settings, but the retail pharmacy coefficient was not statistically significant for women. Conversely, the hospital pharmacy coefficient was not significant for men, but for women, it showed a premium of 8.6%. The coefficients for self-employed pharmacists, the last of the job-related covariates, suggested that both male and female practitioners were heavily penalized for being independent entities—male pharmacists earned 27.7% less, and female pharmacists earned 39.2% less, than their colleagues who worked for an employer.

## 4. Discussion

Three key conclusions may be drawn from the investigation into the pharmacists’ income determination process conducted here. (Two models were compared; in Model 1 gender was measured as a dichotomous covariate in a single equation for all pharmacists, while in Model 2, separate equations were estimated for male and female pharmacists.) First, based on the statistical significance of the least-squares coefficient estimates, the *F* ratios, and the adjusted R^2^ values, both models were successful in describing how earnings were configured. As expected, the work-input covariates (number of hours in an average workweek and number of weeks worked per year) were the predominant determinants of practitioners’ income. Thus, the empirical evidence for 2019–2022 lent evidence to the original specification of the covariates by Goldin and Katz [11], along with their contention that “remuneration is fairly linear with respect to hours of work and weeks” (p. 724). It is nevertheless important to note that the current analysis builds on their results and might not be directly comparable to Goldin and Katz [11] given the different time frame and analysis sample. While Model 1 used the same variables and a similar specification to the one selected by Goldin and Katz [11] in an attempt to examine whether the conclusions they reached held to using a more recent analysis period, the current research also built on their work by extending the analysis to uncover pay gaps along several other dimensions such as marital status, age, race and ethnicity, sector, and employment status.

The second conclusion of this research was that the evidence did not support the assumption of the single-equation specification (Model 1) that the responses of covariates were equal between male and female pharmacists. The male- and female-pharmacist coefficients for average workweek, Black/African American race, Hispanic ethnicity, single/never-married status, and self-employment status had the same sign as in Model 1, but depicted a gap of over 25% in size; in addition, the coefficients for other than White or Black race and working in retail pharmacy were significant for men but not for women, while the coefficient for working in hospital pharmacy was significant for women but not for men. The separate male- and female-pharmacist approach (Model 2) revealed fundamental differences between the genders in how their levels of income are configured.

Third, the empirical results obtained here seem to indicate that the pharmacist annual earnings gender gap is attributable to differences in more than the number of hours worked. According to Table 1, the unadjusted earnings gap of $19,477 per year amounts to women earning 14.7% less than men. If one multiplies the number of hours reported in an average workweek by the number of weeks worked per year, the product yields 2005.4 and 1807.5 h worked per year by male and female practitioners, respectively, that is, a difference of 9.9%, which leaves a 4.8% gap in income unexplained by the number of hours worked per year by men and women. An alternative view would be that male pharmacists earned a wage rate (number of hours worked per year divided into annual income) of $65.96 per hour and female pharmacists earned a wage rate of $62.40 per hour, equivalent to a 5.4% gender wage-rate gap.

Both of these methods to estimate the gender gap, however, underrepresent the extent of the pharmacists’ gender income disparity revealed by Model 2. If one uses the estimated coefficients reported in Table 2 to project the income earned by a Non-Hispanic White, married, 50-year old practitioner with at least one child, who works in retail pharmacy 40 h per week throughout 50 weeks per year, the annual income would be $132,246 if he were a man and $121,503 if she were a woman, that is, a gap of 8.1%. For a Non-Hispanic White, single, 50-year old practitioner with no children, who works in manufacturing or a setting other than retail or hospital pharmacy 40 h per week throughout 50 weeks per year, the projected annual income would be $131,654 if he were a man and $107,149 if she were a woman, showing a gender earnings gap of 18.6%, much greater than originally reported by Goldin and Katz [11] or estimated for 2019–2022 using the single-equation approach of Model 1.

The empirical evidence presented here does not support the conclusion by Goldin and Katz [11] using 2009–2011 data that pharmacy has a low gender earnings gap and is a family-friendly profession. An estimated earnings gap of up to 18.6% (even higher if it applies to self-employed practitioners) is substantial. The estimated earnings gaps endured by Black/African Americans (16.5% for all pharmacists, 19.1% for male pharmacists, and 15.2% for female pharmacists) and Hispanics (15.0% for all pharmacists, 17.4% for male pharmacists, and 13.8% for female pharmacists) are similarly large. Although not directly comparable to Goldin and Katz [11] due to different analysis periods and specifications, these findings suggest that the extent of the racial and ethnic gaps has increased since the publication of their findings.

Non-married practitioners also experienced substantial earnings disparities, although their extent was smaller than the racial and earnings gaps. Single/never-married practitioners earned less income (12.1% for all practitioners, 9.2% for men, and 14.0% for women) than married practitioners, as did separated/divorced/widowed practitioners (9.3% for all practitioners, 9.8% for men, and 9.5% for women). Finally, the greatest earnings gaps were experienced by self-employed pharmacists (33.1% for all pharmacists, 27.7% for men, and 39.2% for women).

## 5. Limitations

The empirical results obtained and reported in this study, and the inferences derived from them, should be interpreted within the narrow analytical scope of the methodology. Accordingly, several limitations ought to be identified. First, this study relied on self-reported survey data, raising concerns about its validity and reliability. While the data were gathered by the U.S. Census Bureau, a reliable source, and the data collection process has been consistently repeated over the years, self-reported data are influenced by the emotions and perceptions of the respondents at the time of the survey; ultimately, the validity of the gender comparison depends on the extent to which the response biases by both genders cancelled each other and the true roots of the income disparities were detected.

Another limitation concerns potential biases from the omission of relevant covariates. The covariates used in the equations were the ones originally identified by Goldin and Katz [11], which are earnings determinants frequently appearing in the literature. The possibility remains, however, that others might have been overlooked.

One also should consider that although the ACS data are longitudinal, this study focused solely on a specific period, namely, 2019–2022. Thus, its application was inadequate to determine if gender-related income disparities, or the way in which the various determinants influence income, change over time, or how these changes are affected by the continuous increase in the number of pharmacy school graduates. For example, if one compares, using the same data source, this study’s findings with the average number of hours worked per week reported for 2009–2011, one notices an increase from 36.6 to 37.1 h for women and a drop from 43.2 to 40.4 h for men; in other words, the weekly gender work-input gap was cut in half, from 6.6 to 3.3 h per week, over a 10-year period as female pharmacists worked more and male pharmacists worked less. Similarly, both the racial and ethnic earnings gaps increased over the last decade.

Another limitation is that the 2019–2022 data used in this study might not have been entirely homogeneous. This period included the COVID-19 pandemic years and may be in some respects atypical of pharmacists’ socioeconomic behavior in the long run. The pandemic had a significant impact on the wages and employment status of pharmacists. The demand for pharmacists increased in response to the surge in healthcare needs [30] and as a result, some pharmacists worked extended hours to meet the demand for services. While wage increases varied by practice setting, job security increased in hospital and clinical settings, while retail pharmacies faced mixed impacts with some staffing reductions. Finally, burnout and stress were high, leading some pharmacists to leave or transition roles [31]. The uniform adjustment of income to 2022 dollars using the CPI was arbitrary and might have overlooked relevant within-period, individual annual variations. It also might have overlooked how income specifically changed from year to year within the period for practitioners in the 2019, 2020, and 2021 sample segments. Furthermore, the levels of income reported by practitioners did not take into consideration spatial variations in taxes and cost of living; both variables are essential in configuring real income, which oftentimes guides socioeconomic behavior more accurately than nominal income.

## 6. Conclusions

Despite its limitations and methodological concerns, this study has served as a vehicle for probing the nature and magnitude of gender disparities in pharmacists’ income and some of its determinants through the utilization of alternative postulates and methodological approaches. It has highlighted the existence of gender disparities wider than previously obtained. It has established the advantage of using separate equations for men and women over a single-equation method that measures gender dichotomously. It also has pointed out the existence of major racial, ethnic, and other income gaps that suggest that pharmacy is only friendly to selected segments of the profession.

Future research is needed to add a longitudinal perspective leading to a better understanding of the evolving role and importance of earnings determinants over time, not only with respect to gender, race, and ethnicity, but also other variables that have yielded significant differences in this study. Hopefully, the arguments and empirical evidence presented here may be used as a stepping stone and generate more research endeavors and policies geared toward making pharmacy truly friendlier to everyone.

## Figures and Tables

**Table 1:** Variable means and standard deviations (in parentheses).

Variable	Means and Standard Deviations		
	Model 1 All Pharmacists	Model 2	
		Male Pharmacists	Female Pharmacists
Number of observations	12,450	4791	7659
ANNUAL INCOME (2022 dollars) [E]	120,292	132,274 *	112,797 *
	(62,863)	(69,904)	(56,759)
GENDER: FEMALE (%) [G]	0.615	0	1
	(0.487)		
WORK-INPUT COVARIATES [W]			
Average workweek (hours)	38.4	40.4 *	37.1 *
	(10.7)	(9.9)	(11.0)
Weeks per year (weeks)	49.1	49.6 *	48.8 *
	(9.5)	(8.6)	(10.0)
HUMAN-CAPITAL COVARIATES [X]			
Age (years)	41.8	42.7 *	41.3 *
	(11.0)	(11.4)	(10.6)
Race: Black/African American (%)	0.051	0.047	0.053
	(0.220)	(0.213)	(0.224)
Race: Other than White or Black (%)	0.288	0.276 ^†^	0.296 ^†^
	(0.453)	(0.447)	(0.456)
Hispanic ethnicity (%)	0.044	0.047	0.042
	(0.204)	(0.212)	(0.200)
Marital status: Single/never married	0.219	0.228	0.214
	(0.414)	(0.420)	(0.410)
Marital status:	0.076	0.065 *	0.083 *
Separated/divorced/widowed (%)	(0.265)	(0.247)	(0.276)
At least one child (%)	0.513	0.496 *	0.523 *
	(0.500)	(0.500)	(0.500)
JOB-RELATED COVARIATES [Z]			
Working in retail pharmacy (%)	0.564	0.594 *	0.545 *
	(0.496)	(0.491)	(0.498)
Working in hospital pharmacy (%)	0.296	0.267 *	0.314 *
	(0.456)	(0.442)	(0.464)
Self-employed (%)	0.034	0.053 *	0.021 *
	(0.180)	(0.225)	(0.144)

* Statistically significant gender differences (*p* ≤ 0.01). ^†^ Statistically significant gender differences (*p* ≤ 0.05).

**Table 2:** Least-squares coefficient estimates, their standard errors (in parentheses), and levels of significance.

Covariate	Estimated Least-Squares Coefficients, Standard Errors, and Levels of Significance			
	Term	Model 1 All Pharmacists	Model 2	
			Male Pharmacists (*i* = 1)	Female Pharmacists (*i* = 2)
Constant term	α*_i_*	6.739333	6.657845	6.656222
Gender: Female	β	−0.061364 *	-	-
		(0.011582)		
Average workweek	γ_1_	0.028897 *	0.024822 *	0.031111 *
		(0.000541)	(0.000923)	(0.000671)
Weeks per year	γ_2_	0.038929 *	0.040682 *	0.037884 *
		(0.000612)	(0.001128)	(0.000728)
Age	λ_1_	0.080543 *	0.089192 *	0.079355 *
		0.005015)	(0.008189)	(0.006431)
Age squared	λ_2_	−0.000818 *	−0.000904 *	−0.000812 *
		(0.000057)	(0.000091)	(0.000074)
Black/African American	λ_3_	−0.180572 *	−0.211494 *	−0.164392 *
		(0.021577)	(0.035562)	(0.027126)
Other than White or Black	λ_4_	−0.020256	−0.048702 ^†^	−0.001379
		(0.012761)	(0.021104)	(0.015987)
Hispanic ethnicity	λ_5_	−0.162511 *	−0.190784 *	−0.148733 *
		(0.025753)	(0.039588)	(0.033946)
Single/never married	λ_6_	−0.128681 *	−0.096558 *	−0.150633 *
		(0.016415)	(0.027024)	(0.020670)
Separated/divorced/widowed	λ_7_	−0.097269 *	−0.103476 *	−0.100044 *
		(0.021430)	(0.038828)	(0.025732)
At least one child	λ_8_	−0.006375	−0.006235	−0.006582
		(0.014106)	(0.023155)	(0.017858)
Working in retail pharmacy	θ_1_	−0.045764 *	−0.085772 *	−0.018335
		(0.016468)	(0.026547)	(0.020970)
Working in hospital pharmacy	θ_2_	0.043463 ^†^	−0.020219	0.082699 *
		(0.017858)	(0.029500)	(0.022410)
Self-employed	θ_3_	−0.401601 *	−0.324638 *	−0.497799 *
		(0.032127)	(0.043238)	(0.048702)
F Statistic		859.4 *	286.0 *	626.2 *
Adjusted R^2^		0.491	0.436	0.515

* Statistically significant (*p* ≤ 0.01). ^†^ Statistically significant (*p* ≤ 0.05).

## Data Availability

Data supporting the results of this study can be found at https://usa.ipums.org/usa/ URL (accessed on 20 May 2024).
